# Contrasting responses of male and female foraging effort to year‐round wind conditions

**DOI:** 10.1111/1365-2656.12419

**Published:** 2015-08-18

**Authors:** Sue Lewis, Richard A. Phillips, Sarah J. Burthe, Sarah Wanless, Francis Daunt

**Affiliations:** ^1^ Institute of Evolutionary BiologySchool of Biological SciencesUniversity of EdinburghEdinburghEH9 3FLUK; ^2^ British Antarctic SurveyNatural Environment Research CouncilHigh CrossMadingley RoadCambridgeCB3 0ETUK; ^3^ Centre for Ecology & HydrologyBush EstatePenicuikEH26 0QBUK

**Keywords:** climate change, demographic rate, environmental perturbation, extreme weather event, *Phalacrocorax aristotelis*, seabird, wind

## Abstract

1. There is growing interest in the effects of wind on wild animals, given evidence that wind speeds are increasing and becoming more variable in some regions, particularly at temperate latitudes. Wind may alter movement patterns or foraging ability, with consequences for energy budgets and, ultimately, demographic rates.
2. These effects are expected to vary among individuals due to intrinsic factors such as sex, age or feeding proficiency. Furthermore, this variation is predicted to become more marked as wind conditions deteriorate, which may have profound consequences for population dynamics as the climate changes. However, the interaction between wind and intrinsic effects has not been comprehensively tested.
3. In many species, in particular those showing sexual size dimorphism, males and females vary in foraging performance. Here, we undertook year‐round deployments of data loggers to test for interactions between sex and wind speed and direction on foraging effort in adult European shags *Phalacrocorax aristotelis*, a pursuit‐diving seabird in which males are *c*. 18% heavier.
4. We found that foraging time was lower at high wind speeds but higher during easterly (onshore) winds. Furthermore, there was an interaction between sex and wind conditions on foraging effort, such that females foraged for longer than males when winds were of greater strength (9% difference at high wind speeds vs. 1% at low wind speeds) and when winds were easterly compared with westerly (7% and 4% difference, respectively).
5. The results supported our prediction that sex‐specific differences in foraging effort would become more marked as wind conditions worsen. Since foraging time is linked to demographic rates in this species, our findings are likely to have important consequences for population dynamics by amplifying sex‐specific differences in survival rates.

## Introduction

Climate change is having a profound effect on the population dynamics of animal species (Walther *et al*. 2002). Whilst much research has focussed on the effects of temperature, changes in wind regimes, particularly an increase in mean wind speeds and storm frequency, are predicted in some regions, notably at temperate latitudes (McInnes, Erwin & Bathols 2011; Young, Zieger & Babanin 2011). Wind may affect demographic rates directly (Moreno & Moller 2011), or indirectly by influencing the ability to forage, including the costs of travelling to suitable habitats, and efficiency in terms of pursuing and capturing prey (Daunt *et al*. 2006; Weimerskirch *et al*. 2012). These effects are apparent in atmospheric and terrestrial environments, as well as aquatic environments because of the profound effects of wind on wave patterns (Chapman *et al*. 2011).

The effects of weather on foraging dynamics are unlikely to be equal for all individuals (Coulson *et al*. 2006). Understanding how individuals vary in their response is of major importance, since increased wind speeds in the future could result in greater variation in demographic rates, with profound consequences for population dynamics. Numerous studies have demonstrated differences in foraging performance between the sexes across a range of taxa, usually, but not always, associated with sexual size dimorphism (Schoener 1967; Le Boeuf *et al*. 2000; Lewis *et al*. 2002). Much of the research has been undertaken on birds, in particular seabirds, providing evidence that males and females differ in competitive ability, flight or foraging efficiency (e.g. Weimerskirch *et al*. 1997; Phillips *et al*. 2004). Most studies have found that the larger sex has a higher foraging efficiency and, consequently, a lower average foraging effort to attain its daily energy requirements. These differences can lead to sex‐specific variation in demographic rates (Jaeger *et al*. 2014). However, the influence of environmental conditions such as wind speed and direction on sex differences in foraging effort or efficiency has not been investigated in detail. There is a general prediction that variation in performance among individuals (due to sex, age or other intrinsic factors) will become more marked as environmental conditions worsen (e.g. Sydeman *et al*. 1991). Accordingly, we might expect sex‐specific differences in foraging effort or efficiency to be amplified as wind conditions deteriorate. For the many species that forage by diving, increased wind speed affects foraging effort because cost of travel increases with greater turbulence in the water column, and foraging efficiency declines because of the difficulty in capturing prey (Finney, Wanless & Harris 1999; Dehnhard *et al*. 2013). Wind direction is also important since wave action is determined by the interaction between wind and bathymetry (Mann & Lazier 1996). Thus, sex‐specific foraging effort is predicted to be more marked at high wind speeds and when wind direction is perpendicular to rising bathymetry (e.g. onshore winds in exposed coastal environments, Daunt *et al*. 2006).

Here, we test the interaction between sex and wind on foraging effort in the sexually size‐dimorphic European shag *Phalacrocorax aristotelis* (hereafter ‘shag’). The shag is a coastal, piscivorous seabird that obtains prey by pursuit‐diving (Watanuki *et al*. 2008). We collected data on time spent foraging (hereafter ‘foraging time’) over a 24‐month period by attaching data loggers to adults of both sexes breeding at a colony in south‐east Scotland. We consider this to be a useful study system for several reasons. First, the effects of wind on diving species are likely to be particularly marked in coastal locations with limited refuges (Mann & Lazier 1996), such as the east coast of the UK. Second, Europe is among the regions considered most likely to see future increases in mean and maximum wind speeds (McInnes, Erwin & Bathols 2011). Third, there are links between wind conditions, foraging time and demographic rates in the study population, specifically (i) survival rates are lower and subsequent timing of breeding, a strong determinant of breeding success, later when onshore winds in late winter are more frequent (Aebischer 1986; Aebischer & Wanless 1992; Frederiksen *et al*. 2008); (ii) survival rates and subsequent breeding success are reduced in years in which winter foraging time of adults is high, suggesting there is a link between foraging effort and demography (Daunt *et al*. 2014); (iii) foraging time is affected by wind such that it increases during strong onshore winds and decreases at high wind speeds (Daunt *et al*. 2006, 2014). Crucially, the interaction between sex and wind on foraging time has not been tested. We controlled for a suite of other factors that may affect sex‐specific foraging time, including those that may arise from division of labour during reproduction (Elliott, Gaston & Crump 2010). We accounted for autocorrelation at the individual and population level, since it can influence effect sizes (Fieberg *et al*. 2010). Our approach enabled us to test the prediction that there would be a sex by wind interaction, such that foraging time of females would be progressively longer than that of males as wind conditions deteriorate.

## Materials and methods

### Study Site and Data Collection

The study was carried out on the Isle of May National Nature Reserve, south‐east Scotland (56°11′N, 02°33′W). During chick rearing in the 2009 breeding season, 88 adult shags were fitted with combined Global Location Sensing (GLS)/immersion loggers (MK7, MK13 and MK14; British Antarctic Survey; 20 × 9 × 5·5–22 × 19 × 4 mm; mass 1·5–3·0 g, *c*. 0·1–0·2% body mass), attached to a plastic leg ring with cable ties. Similar loggers representing 0·23% of body mass did not significantly affect diving ability in the closely related great cormorant *P. carbo* (Ropert‐Coudert *et al*. 2009). Laying date (recorded directly, or back‐calculated from hatching dates) and breeding success (number of chicks fledged per pair) were recorded from frequent nest checks, and individuals sexed on voice, size and behaviour (*n* = 57 males and 31 females). Birds were resighted in the 2010 breeding season; 75 were caught, their body mass measured to the nearest 10 g with a Pesola spring balance, the logger removed and a new device deployed (73 complete data sets i.e. 97·3%; 49 males, 25 females; body mass: males 1928·4 g ± 134·9 SD, females 1636·7 g ± 113·2 SD; males on average 17·8% heavier). Loggers were deployed on 24 new individuals of known laying date during chick rearing in 2010 (*n* = 16 males and 8 females) to maximise the chances that sample sizes would be comparable in 2009/2010 and 2010/2011, and their breeding success recorded. Birds were resighted again in 2011 and 72 loggers were retrieved (100% complete data sets; *n* = 47 males; *n* = 25 females; body mass: males 1935·0 g ± 128·1 SD; females 1616·8 g ± 109·5 SD; males on average 19·7% heavier), and laying date and breeding success recorded. The complete sample comprised 96 birds (64 males, 32 females; 49 birds with data for 2009–2011, 24 for 2009–2010, 23 for 2010–2011). Handling times were <5 min, and we recorded no negative effects of device deployment or retrieval.

### Data Processing

The loggers detect immersion in sea water, recorded every 3 s. Because shags have a wettable plumage, time spent in the water is a reliable proxy of foraging time (*r* = 0·94, *n* = 48 individuals, *P* < 0·001; Daunt *et al*. 2007a). Shags spend the night on land, providing a natural break in foraging, so we calculated daily foraging time (h). We obtained hourly wind data from Leuchars weather station (56°23′N, 02°52′W, 26 km north of the colony; www.badc.nerc.ac.uk) and calculated daily mean wind speed and direction (following Daunt *et al*. 2006, 2014). The population is partially migratory with breeding adults recorded 486 km to the north and 136 km to the south of the colony in the nonbreeding period (Grist *et al*. 2014). The geolocation data were too imprecise to provide accurate locations for study individuals; however, wind speeds and directions were strongly correlated across this distribution (see Appendix S1), suggesting that individuals experienced similar conditions.

### Model Selection

The modelling was undertaken in two steps. First, the most appropriate explanatory variables to include in the model were determined. The relationship between daily foraging time and explanatory variables was modelled with a linear mixed model using restricted maximum likelihood with a Gaussian error distribution (Zuur *et al*. 2009). Models included individual as a random effect to account for nonindependence of observations. We fitted sex (two level factor), mean daily wind speed and mean daily wind direction (two level factor, east or west of north) as fixed effects. We also included date (days after 1st June) to account for within‐year variation. We have shown previously that the relationship between foraging time and date is quadratic in the nonbreeding period (Daunt *et al*. 2006, 2007a, 2014). Here, we modelled the full annual cycle, but visual inspection of the data suggested that peak foraging time occurred in winter so we fitted quadratic and linear date, as in earlier studies based on restricted periods of the annual cycle. Year was fitted as a two level factor (Year 1 = 01/06/09–31/05/10; Year 2 = 01/06/10–31/05/11) to account for the average population‐level effect of year. We also included the effect of breeding status as a two level factor, breeding comprising the period from laying until offspring independence at age 90 days (total 126 days) and nonbreeding comprising the remainder of the annual cycle (Daunt *et al*. 2007a). For the breeding period, we included days since laying and, from hatching to offspring independence, we included brood size (range: 1–4), since both are known to influence foraging effort (Wanless, Harris & Russell 1993; Gremillet *et al*. 2005). Visual inspection showed that foraging time in relation to days since laying was cubic, and so we fitted a cubic function. Individuals that failed in breeding (*n* = 4) were excluded until the point their chicks would have been independent (i.e. age 90 days). We fitted linear and quadratic adult age to account for age‐specific effects (Daunt *et al*. 2007b; Lecomte *et al*. 2010). Individuals were either of known age if ringed as chicks (*n* = 91) or assumed to have been aged 3 at ringing if ringed as adults (following Grist *et al*. 2014; *n* = 5). All covariates were standardised by subtracting the mean and dividing by one standard deviation (Zuur *et al*. 2009; Schielzeth 2010). Two‐way interactions of sex by wind speed and sex by wind direction were fitted to test the principal aim of this paper. We also fitted sex by linear date, quadratic date, breeding status and brood size to test whether sex differences were accentuated when conditions are more challenging. We fitted all fixed effects to every model, and tested all possible combinations of two‐way interaction terms (*n* = 48; Appendix S2). Model selection was performed using Akaike information criterion (AIC_c_), where the best model had the lowest AIC_c_ value. Models within two AIC (ΔAIC < 2) were considered to have equal support (Burnham & Anderson 2002), unless they contained one or more parameter and had a higher AICc than the best supported model, where this rule of thumb is not considered appropriate (Burnham & Anderson 2002). Model selection was performed using the nlme and MuMIn packages in r, version 3.0.1 (R Core Team, www.R-project.org).

In a second step, we fitted a series of alternative random effects structures to test the importance of individual‐ and population‐level autocorrelation between consecutive days and sex‐specific repeatability. This analysis was undertaken in the ASReml package using r, version 3.0.1 (R Core Team, www.R-project.org) (see Appendix S3 for full details).

## Results

The best supported model included interaction terms between sex and wind speed, wind direction, breeding status and brood size (see Appendix S2 for candidate model set).

### Sex and Wind Effects on Foraging Time

**Figure 1:**
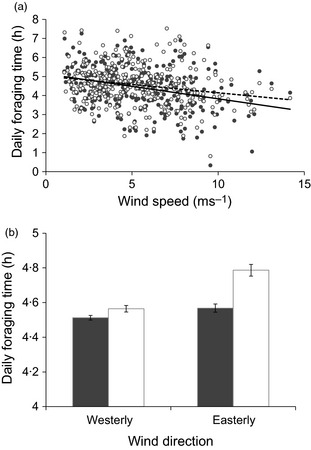
(a) Mean daily foraging time (h) by wind speed (ms^−1^) and sex (males – closed circles with solid line; females – open circles with dashed line) for shags on the Isle of May from 2009 to 2011; (b) daily foraging time (h) ± standard errors by wind direction (westerly; easterly) and sex (males – closed bars; females – open bars) for shags on the Isle of May from 2009–2011.

Daily foraging time decreased with increasing wind speed, and this reduction was more pronounced in males than females (sex by wind speed interaction, Table 1, Fig. 1a; difference between females and males: 8·6% for wind speed of 14 ms^−1^; 1·4% for wind speed of 1 ms^−1^). Individuals foraged for longer per day during easterly winds, and the difference between males and females was more marked during these conditions (sex by wind direction interaction, Table 1; Fig. 1b; difference between females and males: 7·0% for easterly winds; 4·0% for westerly winds).

**Table 1:** Estimates (est) ± standard errors (SE), variance components ± SE and *z* ratios from our final model of daily foraging time in shags on the Isle of May from 2009 to 2011. The intercept corresponds to the mean value for males during the breeding season in 2009 when wind was in an easterly direction. The random effects model is described in Appendix S3

Fixed effects	Est	SE	*z* ratio
Intercept	4·788	0·213	22·506
Sex (female)	0·430	0·129	3·336
Wind speed	−0·198	0·026	−7·588
Wind direction (W)	−0·069	0·055	−1·264
Year (2010–11)	−0·438	0·176	−2·486
Date	−0·164	0·081	−2·020
Date^2^	−0·962	0·102	−9·447
Breeding status (non‐breeding)	0·860	0·107	8·011
Brood size	0·357	0·032	11·134
Days since laying	−0·200	0·116	−1·732
Days since laying^2^	1·560	0·131	11·904
Days since laying^3^	−0·498	0·035	−14·070
Age	−0·172	0·046	−3·730
Age^2^	−0·017	0·024	−0·727
Wind speed*sex (F)	0·090	0·017	5·452
Wind direction (W)*sex (F)	−0·123	0·036	−3·426
Breeding status (non‐breeding)*sex (F)	−0·388	0·089	−4·379
Brood size*sex (F)	−0·103	0·059	−1·754

Random effects	Variance component	SE	*z* ratio
Variance of individual effects	0·186	0·029	6·368
Individual correlation	0·316	0·004	83·393
Population variance	0·799	0·078	10·274
Population correlation	0·760	0·022	33·836
Residual variance	2·720	0·019	145·785

### Other Factors Affecting Foraging Time

**Figure 2:**
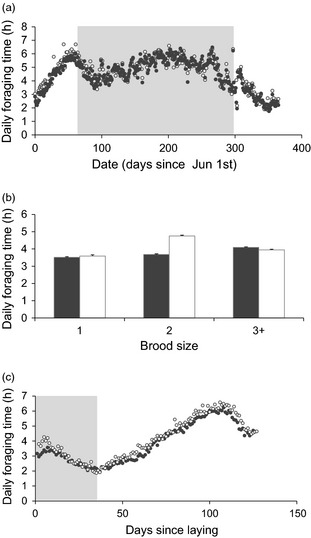
(a) Mean daily foraging time (h) by date (days since June 1st) and sex (males closed circles; females open circles) for shags on the Isle of May from 2009 to 2011. The shaded box shows the winter period (from offspring independence to the onset of laying in the following breeding season); (b) Mean daily foraging time (h) ± standard errors in relation to brood size and sex (males closed bars; females open bars); (c) Mean daily foraging time (h) in relation to days since laying (days) and sex (males closed circles; females open circles); the shaded box shows incubation (days 1–36).

There was a linear and quadratic effect of date on daily foraging time, which rose to a peak in midwinter, before declining in the spring (Fig. 2a). However, there was little evidence that males and females showed differing patterns of change with date (Appendix S2). Daily foraging time was lower in the second year of the study and declined with linear age (Table 1). Daily foraging time was greater during the nonbreeding season than the breeding season, and there was a sex by breeding status interaction, with females only foraging more than males during breeding (male: breeding 3·84 ± 0·02; nonbreeding 4·90 ± 0·01; female: breeding 4·16 ± 0·03; nonbreeding 4·88 ± 0·02; Table 1). Foraging times increased with brood size, and there was a sex by brood size interaction such that foraging time of females increased more rapidly than that of males with increasing brood size (Table 1, Fig. 2b). Foraging time showed a cubic relationship with days since laying, decreasing during incubation, increasing from hatching until chicks were *c*. 70 days old, and then declining (Fig. 2c).

## Discussion

### Sex and Wind Effects on Foraging Time

Sex‐specific foraging performance is a widespread phenomenon among animal species (Schoener 1967; Le Boeuf *et al*. 2000; Lewis *et al*. 2002). However, the extent to which this varies in response to environmental conditions is largely unexplored. Here, we provide the first comprehensive evidence of an interaction between sex and wind conditions on foraging effort. This interaction is broadly analogous to those between environmental conditions and age on foraging or breeding performance (e.g. Sydeman *et al*. 1991; Daunt *et al*. 2007b). Foraging time was higher during onshore winds but lower at high wind speeds, which suggests that increased wave action reduces prey capture rates such that it becomes less economical to continue feeding, hence individuals return sooner to land (Daunt *et al*. 2006). Crucially, females had higher foraging times than males when winds were onshore and of greater strength. Thus, differences in foraging effort among the two sexes become more marked as conditions deteriorate, and females are less likely to sit out the poorest conditions. Without more detailed data on diving performance or direct estimates of foraging efficiency, the precise mechanisms driving sex differences in the relationship between wind and foraging effort are unknown. However, one possibility is that females have a lower foraging efficiency than males because their smaller body mass reduces breath‐hold capacity and hence maximum diving depth. Another potential explanation is that the two sexes may differ in flight efficiency due to differences in body mass and wing loading (Wakefield *et al*. 2009), such that males are less buffeted by rapid changes in air currents, which may affect daily energy requirements and therefore the foraging effort required to meet these costs. To test these assertions, future studies could focus on energetic differences between the two sexes.

### Other Factors Affecting Foraging Time

Although foraging time was higher in the nonbreeding period, likely due to the more challenging conditions including reduced prey availability (Daunt *et al*. 2006, 2014), foraging time of females was greater than that of males in the breeding but not the nonbreeding season. This would appear counter to our assertion that sex differences will be accentuated when conditions are poorer. However, as diurnal foragers, shags are constrained by day length in mid‐winter, and thus, the variation among individuals reduces considerably at this time of the year (Daunt *et al*. 2006, 2014). These seasonal patterns are consistent with the suggestion that foraging efficiency may differ between the sexes, and females can increase foraging time to compensate for poorer efficiency when less constrained by day length (Daunt *et al*. 2007a). During the breeding season, foraging time was lower in incubation than chick rearing, with the pattern over the complete breeding cycle exhibiting a striking, cubic form (Gremillet *et al*. 2005), and there was an increase in foraging time with brood size. The difference in foraging time between the two sexes became more marked with increasing brood size, which suggests that females were more affected by the higher energetic demands associated with provisioning of more offspring, although it is not clear why foraging time of adults with broods of two was higher on average that adults with broods of three.

### Demographic Consequences

Previous work on this population has shown that shags experience high mortality during prolonged onshore winds (Frederiksen *et al*. 2008), and that females have lower survival rates than males during such periods (survival during winter 2012/13: females: 0·45, 0·41–0·50 95% CI; males: 0·55, 0·50–0·60; survival during 2009/10 to 2011/12: female mean across years: 0·97; male mean: 0·98; S.J. Burthe, unpublished data). Thus, it is possible that any increase in the frequency of extreme weather events, as predicted by climate models for this region (McInnes, Erwin & Bathols 2011), may result in increasing divergence in survival rates between the sexes. If this is the case, the consequences for effective population size are likely to be profound. Furthermore, this situation could be apparent in the many other species which exhibit sex differences in foraging, and potentially also in demographic rates (Jaeger *et al*. 2014), as environmental perturbations are forecast to become more frequent in many regions. However, the overall effects of future climate change on population size are likely to be complex. There could be benefits to increased wind speeds, if they enhance productivity through coastal upwelling (Mann & Lazier 1996), and the balance of costs and benefits of increased wind speed may shape changes in sex‐specific survival rates. In addition, the direction of the sex effect may switch depending on the demographic rate. For example, reductions in food availability or increases in parasite load may differentially impact survival of male chicks (Reed *et al*. 2008), which may offset any sex‐specific wind effects on adult survival.

### Implications of Future Climate Change

Whilst much of the research on the impacts of climate change on wild animals has focussed on temperature, the effects of wind are an under‐studied but potentially important driver of foraging performance and winter survival probability in some environments (Fort, Porter & Gremillet 2009). Here, we provide evidence that divergence in sex‐specific foraging performance becomes more marked as wind conditions become more severe. This phenomenon may be widespread, given the many studies that have demonstrated sex‐specific differences in foraging performance, although the effects may differ among species due to differences in morphology or environments experienced, with coastal species in exposed locations potentially the most vulnerable. Further studies that quantify the link between sex‐specific foraging performance and demographic rates are needed in order to predict the consequences of changing future wind patterns on population dynamics.

## Data accessibility

Data available from the Dryad Digital Repository: http://dx.doi.org/10.5061/dryad.05pk3 (Lewis *et al*. 2015).

## Supporting information


**Appendix S1.** Wind conditions across the winter range.
**Fig S1.1.** Map showing the Isle of May (large circle), sites where shags were observed in winters 2009–2012 (numbered small circles; Grist *et al*. 2014) and four coastal weather stations (stars).
**Fig S1.2**. Relationship between daily mean sine wind direction at Leuchars weather station and at (a) Lossiemouth weather station; (c) Peterhead weather station; (e) Boulmer weather station; relationship between daily mean wind speed at Leuchars weather station and at (b) Lossiemouth weather station; (d) Peterhead weather station; (f) Boulmer weather station.
**Appendix S2**. Model selection.
**Table S2.1.** Model selection tables (*n* = 48), ordered by AIC_c_.
**Appendix S3**. Within‐individual and population correlations.
**Table S3.1.** Variance components ± standard errors (SE) and *z* ratio for the different random effect models and the likelihood ratio tests (LRTs) comparing the fit of models on daily foraging time in shags on the Isle of May from 2009–2011.
