# Supplementary material for: Contrasting responses of male and female foraging effort to year‐round wind conditions
Source: J Anim Ecol. 2015 Aug 18;84(6):1490–6. doi: 10.1111/1365-2656.12419 (PMC4989534; doi:10.1111/1365-2656.12419)
Supplement: Supplementary file 1 — Appendix S1. Wind conditions across the winter range. Fig S1.1. Map showing the Isle of May (large circle), sites where shags were observed in winters 2009–2012 (numbered small circles; Grist et al. 2014) and four coastal weather stations (stars). Fig S1.2. Relationship between daily mean sine wind direction at Leuchars weather station and at (a) Lossiemouth weather station; (c) Peterhead weather station; (e) Boulmer weather station; relationship between daily mean wind speed at Leuchars weather station and at (b) Lossiemouth weather station; (d) Peterhead weather station; (f) Boulmer weather station. Appendix S2. Model selection. Table S2.1. Model selection tables (n = 48), ordered by AIC c. Appendix S3. Within‐individual and population correlations. Table S3.1. Variance components ± standard errors (SE) and z ratio for the different random effect models and the likelihood ratio tests (LRTs) comparing the fit of models on daily foraging time in shags on the Isle of May from 2009–2011. [file JANE-84-1490-s001.pdf]

Appendix S1: Wind conditions across the winter range

To analyse the effects of wind speed and direction on foraging time, we obtained hourly wind data from Leuchars weather station (56° 23'N, 02° 52'W, 26km to the north of the breeding colony; www.badc.nerc.ac.uk). The population is partially migratory with a winter distribution spanning 486 km linear coastal distance to the north and 136 km linear coastal distance to the south of the Isle of May (Grist *et al.* 2014). The locational data recorded by the geolocation loggers are not sufficiently precise to provide accurate locations for study individuals (Phillips *et al.* 2004). Thus, individuals may have been distributed across the winter range, so we tested whether the wind conditions at Leuchars weather station were representative of the winter range as a whole.

We calculated the correlation between daily mean wind direction (sine-transformed to remove circularity) at Leuchars with daily mean wind direction recorded at three other coastal weather stations distributed approximately evenly across the wintering range, two to the north (Lossiemouth, 57° 42'N, 03° 18'W, 386 km linear coastline distance from the Isle of May; Peterhead, 57° 30'N, 01° 46'W, 247 km linear coastline distance from the Isle of May) and one to the south (Boulmer, 55° 25'N, 01° 35'W, 108 km linear coastline distance from the Isle of May; Fig S1.1; wind data from www.badc.ac.uk; shags do not typically cross land or long stretches of open sea so coastline distance is more biologically relevant than Euclidean distance, Grist et al. 2014). We also correlated daily mean daily wind speed at Leuchars with daily mean wind speed from the other three weather stations. The correlations were calculated for 01/10/09-31/03/10 and 01/10/10-31/03/11, which incorporate the two migratory periods in the study (Grist *et al.* 2014).

The Pearson correlation coefficients between sine wind direction at Leuchars and Lossiemouth, Peterhead and Boulmer were, respectively 0.85, 0.85 and 0.85 (Fig S1.2 a, c

and e). The Pearson correlation coefficients between wind speed at Leuchars and Lossiemouth, Peterhead and Boulmer were, respectively 0.66, 0.64 and 0.78 (Fig S1.2b, d and f).

References

Grist, H., Daunt, F., Wanless, S., Nelson, E.J., Harris, M.P., Newell, M., Burthe, S. & Reid, J.M. (2014) Site fidelity and individual variation in winter location in partially migratory European shags. *Plos One,* **9,** e98562.

Phillips, R.A., Silk, J.R.D., Croxall, J.P., Afanasyev, V. & Briggs, D.R. (2004) Accuracy of geolocation estimates for flying seabirds. *Marine Ecology Progress Series,* **266,** 265-272.

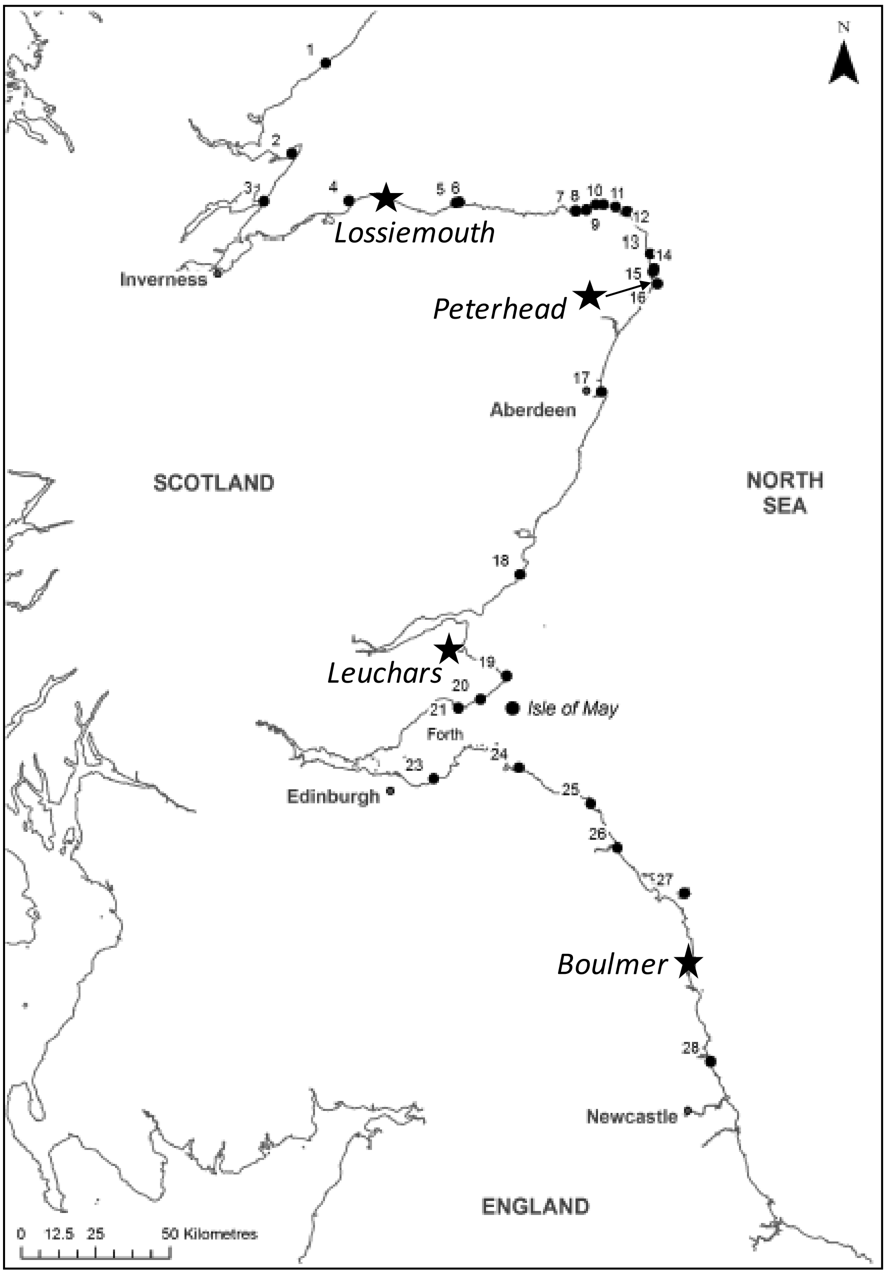

Fig S1.1 Map showing the Isle of May (large circle), sites where shags were observed in winters 2009-2012 (numbered small circles; Grist et al. 2014) and four coastal weather stations (stars).

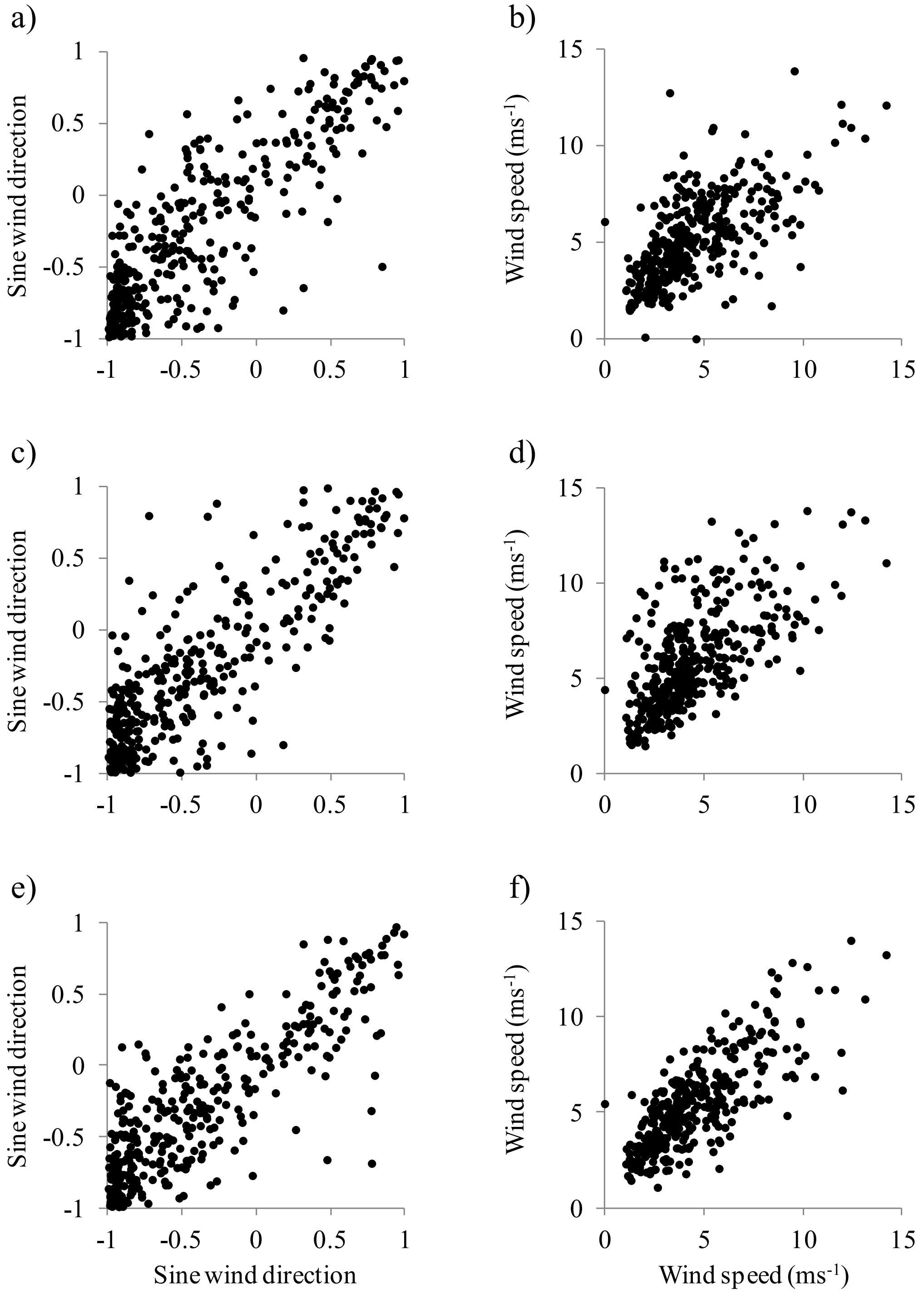

Fig S1.2. Relationship between daily mean sine wind direction at Leuchars weather station and at a) Lossiemouth weather station; c) Peterhead weather station; e) Boulmer weather station; relationship between daily mean wind speed at Leuchars weather station and at b) Lossiemouth weather station; d) Peterhead weather station; f) Boulmer weather station.

Appendix S2: Model selection

The full fixed effects model was as follows:

daily foraging time (h) = sex+wind speed+wind direction+year+date+date^2+breeding status+ brood size+days since laying+days since laying^2+days since laying^3+age+age^2+sex*(wind speed+wind direction+date+date^2+breeding status+brood size); random model = individual

All covariates were standardised by subtracting the mean and dividing by one standard deviation (Zuur *et al.* 2009; Schielzeth 2010). We fitted all fixed effects to every model, and tested all possible combinations of two-way interaction terms (n=48; Appendix S2). Model selection was performed using Akaike Information Criterion (AICc), where the best model had the lowest AICc value. Models within two AICc (ΔAICc<2) were considered to have equal support (Burnham & Anderson 2002), unless they contained one or more parameters and had a higher AICc than the best supported model, where this rule of thumb is not considered appropriate (Burnham & Anderson 2002). Model selection was performed using the *nlme* and *MuMIn* statistical packages in the program R (version 3.0.1).

The best supported model included all fixed effects and interaction terms between sex and wind speed, wind direction, breeding status and brood size (Table S2.1). Two other models were within two AICc (ΔAICc<2) of this model, containing the same effects plus sex by date, and the same effects plus sex by date and sex by date^2, respectively. However, both models were not considered to have equal support as the top model because they contained one or more parameters and had a higher AICc (Burnham & Anderson 2002). These model outputs therefore suggest limited support for a sex by date or sex by date^2 interaction.

References

Burnham, K.P. & Anderson, D.R. (2002) *Model Selection and Multimodel Inference: A Practical Information-Theoretic Approach,* 2nd edn. edn. Springer, New York.

Schielzeth, H. (2010) Simple means to improve the interpretability of regression coefficients. *Methods in Ecology and Evolution,* **1,** 103-113.

Zuur, A.F., Ieno, E.N., Walker, N., Saveliev, A.A. & Smith, G.M. (2009) *Mixed Effects Models and Extensions in Ecology with R*. Springer, New York.

Table S2.1. Model selection tables (n=48), ordered by AICc. The models within two AICc (ΔAICc<2) of the model with the lowest AIC are shown in bold

model	sex interactions added to the main effects model	AICc	ΔAICc
**41**	**sex*wind speed+sex*wind direction+sex*breeding status+sex*brood size**	**207919**	**0.0**
**46**	**sex*wind speed+sex*wind direction+sex*date+sex*breeding status+sex*brood size**	**207920**	**0.6**
**47**	**sex*wind speed+sex*wind direction+sex*date+sex*date^2+sex*breeding status+sex*brood size**	**207920**	**0.6**
27	sex*wind speed+sex*breeding status+sex*brood size	207928	9.2
37	sex*wind speed+sex*date+sex*breeding status+sex*brood size	207928	9.3
42	sex*wind speed+sex*date+sex*date^2+sex*breeding status+sex*brood size	207930	11.2
28	sex*wind direction+sex*breeding status+sex*brood size	207943	23.9
38	sex*wind direction+sex*date+sex*breeding status+sex*brood size	207944	25.2
43	sex*wind direction+sex*date+sex*date^2+sex*breeding status+sex*brood size+sex*brood size	207946	27.5
44	sex*wind speed+sex*wind direction+sex*date+sex*date^2+sex*breeding status	207948	29.0
39	sex*wind speed+sex*wind directionsex*date+sex*breeding status	207949	29.9
11	sex*breeding status+sex*brood size	207949	30.3
29	sex*wind speed+sex*wind directionsex*breeding status	207949	30.5
21	sex*date+sex*breeding status+sex*brood size	207950	31.2
31	sex*date+sex*date^2+sex*breeding status+sex*brood size	207954	34.7
22	sex*wind speed+sex*date+sex*breeding status	207957	38.5
12	sex*wind speed+sex*breeding status	207959	39.6
32	sex*wind speed+sex*date+sex*date^2+sex*breeding status	207959	39.6
36	sex*wind speed+sex*wind direction+sex*date+sex*date^2	207973	54.1
13	sex*wind direction+sex*breeding status	207974	54.6
23	sex*wind directionsex*date+sex*breeding status	207974	54.8
45	sex*wind speed+sex*wind direction+sex*date+sex*date^2+sex*brood size	207974	54.8
33	sex*wind direction+sex*date+sex*date^2+sex*breeding status	207975	56.4
7	sex*date+sex*breeding status	207980	60.6
26	sex*wind speed+sex*wind direction+sex*date	207980	60.7
2	sex*breeding status	207980	60.9
17	sex*date+sex*date^2+sex*breeding status	207983	63.6
19	sex*wind speed+sex*date+sex*date^2	207983	63.8
34	sex*wind speed+sex*date+sex*date^2+sex*brood size	207983	64.3
16	sex*wind speed+sex*wind direction	207986	67.4
40	sex*wind speed+sex*wind direction+sex*date+sex*brood size	207987	68.1
30	sex*wind speed+sex*wind direction+sex*brood size	207993	74.3
9	sex*wind speed+sex*date	207995	75.7
35	sex*wind direction+sex*date+sex*date^2+sex*brood size	207995	76.1
20	sex*wind direction+sex*date+sex*date^2	207996	76.7
24	sex*wind speed+sex*date+sex*brood size	208001	82.5
18	sex*date+sex*date^2+sex*brood size	208002	82.7
6	sex*date+sex*date^2	208002	83.4
4	sex*wind speed	208003	83.8
10	sex*wind direction+sex*date	208004	84.6
14	sex*wind speed+sex*brood size	208009	89.8
5	sex*wind direction	208009	90.2
25	sex*wind direction+sex*date+sex*brood size	208011	92.1
1	sex*date	208015	96.2
15	sex*wind direction+sex*brood size	208016	97.3
0		208022	103.1
8	sex*date+sex*brood size	208022	103.2
3	sex*brood size	208028	109.4

Appendix S3: Within-individual and population correlations

Methods

We fitted a series of alternative random effects structures to test the importance of individual and population level auto-correlation between consecutive days and sex-specific repeatability. This analysis was undertaken in the *asreml* statistical package in the program R (version 3.0.1).

The first model (model 1) contained individual identity to account for consistent effects of an individual in average daily foraging time. This term was equivalent to the random term fitted in the first modelling step using AIC (Appendix S2). We also fitted the correlation with an individual's foraging time on the previous day (see 'individual correlation' in Table 1 in main text and in Table S3.1), because the foraging behaviour of an individual on consecutive days may be expected to be more similar than on non-consecutive days (because of temporal autocorrelation in environmental conditions) and the residual variance. The covariance of foraging time between successive days within individuals was calculated by multiplying the correlation by the residual variance. We also fitted the correlation between days (see 'population correlation' in Table 1 in main text and in Table S3.1) to measure the correlation between the average daily foraging time of all individuals between successive days (to examine whether all individuals were responding in the same way to between-day effects due to e.g. shared environmental conditions) and the variance of the population's daily foraging time (see 'population variance' in Table 1 in main text and in Table S3.1). The covariance of the average daily foraging time across the population between successive days was calculated by multiplying the correlation and the population variance. The total correlation between successive foraging times (sometimes referred to as the temporal

correlation) was then calculated from the sum of the within and between individual covariances, divided by the total variance.

To assess the importance of the individual correlation, we carried out a second model (model 2) excluding this term from model 1. Similarly, to assess the importance of the population correlation we carried out a third model (model 3) excluding this term from model 1. To test whether individual consistency varied in strength with sex, we carried out a fourth model (model 4), which was the same as model 1 except the individual random term was split between the sexes. Repeatability for each sex was estimated by dividing the sex specific variance by the total variance (e.g., male repeatability: variance of individual effects (male) / variance of individual effects (male) + population variance + residual variance).

Models 2-4 were each compared with model 1 using a *likelihood-ratio test* (LRT) with 1 df to assess whether models were significantly different on removing particular random components (Zuur *et al.* 2009). A significant difference between the models would indicate that the term removed was important, and should therefore be retained. In all of the random models, days were coded as days since June 6th 2009, when sampling began, running continuously until the end of sampling on 31st May 2011. Detailed model descriptions are as follow:

model 1

daily foraging time (h) = sex+wind speed+wind direction+year+date+date^2+breeding status+ brood size+days since laying+days since laying^2+days since laying^3+age+age^2+sex*(wind speed+wind direction+breeding status+brood size); random model = variance of individual effects (individual) + individual correlation + population correlation + population variance + residual variance

model 2

daily foraging time (h) = sex+wind speed+wind direction+year+date+date^2+breeding status+ brood size+days since laying+days since laying^2+days since laying^3+age+age^2+sex*(wind speed+wind direction+breeding status+brood size); random model = variance of individual effects (individual) + population correlation + population variance + residual variance

model 3

daily foraging time (h) = sex+wind speed+wind direction+year+date+date^2+breeding status+ brood size+days since laying+days since laying^2+days since laying^3+age+age^2+sex*(wind speed+wind direction+breeding status+brood size); random model = variance of individual effects (individual) + individual correlation + population variance + residual variance

model 4:

daily foraging time (h) = sex+wind speed+wind direction+year+date+date^2+breeding status+ brood size+days since laying+days since laying^2+days since laying^3+age+age^2+sex*(wind speed+wind direction+breeding status+brood size); random model = variance of individual effects (male) + variance of individual effects (female) + individual correlation + population correlation + population variance + residual variance

Results

Table S3.1 shows the variance components and standard errors of the different random models (models 1-4). The covariance of foraging time between successive days within individuals was high (0.86) and the effect was highly significant (delta LL =2626, $p<0.001$; Table S3.1). The covariance of the average daily foraging time across all individuals (population) between successive days was also high (0.61) and highly significant (delta LL =254, $p<0.001$; Table S3.1). The total correlation between successive foraging times, or temporal correlation, was 0.40. There was no evidence that accounting for sex specific variation in the random effects structure of model 4 was important (delta LL =0.8, $p=1.0$; Table S3.1), suggesting that the sexes did not differ in their overall variance. In line with this, the overall repeatability of male and female daily foraging time was similar (male: 0.044; female: 0.063). Thus, model 1 was favoured and is presented in Table 1 in the main text.

References

Zuur, A.F., Ieno, E.N., Walker, N., Saveliev, A.A. & Smith, G.M. (2009) *Mixed Effects Models and Extensions in Ecology with R*. Springer, New York.

Table S3.1. Variance components ± standard errors (s.e.) and z ratio for the different random effect models and the likelihood ratio tests (LRTs) comparing the fit of models on daily foraging time in shags on the Isle of May from 2009-2011.

**model**	**random effects:**	**variance component**	**s.e.**	**z ratio**
**1**	variance of individual effects	0.186	0.029	6.368
	individual correlation	0.316	0.004	83.393
	population variance	0.799	0.078	10.274
	population correlation	0.760	0.022	33.836
	residual variance	2.720	0.019	145.785
**2**	variance of individual effects	0.196	0.030	6.567
	population variance	0.797	0.082	9.747
	population correlation	0.783	0.022	35.632
	residual variance	2.717	0.017	158.300
**3**	variance of individual effects	0.186	0.029	6.362
	individual correlation	0.317	0.004	83.760
	population variance	0.686	0.039	17.634
	residual variance	2.725	0.019	145.522
**4**	variance of individual effects (male)	0.160	0.031	5.173
	variance of individual effects (female)	0.239	0.064	3.707
	individual correlation	0.316	0.004	83.392
	population variance	0.799	0.078	10.273
	population correlation	0.761	0.022	33.839
	residual variance	2.720	0.019	145.786

**model**		**log Likelihood**	**delta LL**	**p (chisq)**
**1**	Main model	-49404.570	*	*
**2**	Testing individual correlation	-52031.180	2626.610	0.000
**3**	Testing population correlation	-49658.220	253.650	0.000
**4**	Comparing sexes	-49403.820	0.750	1.000
